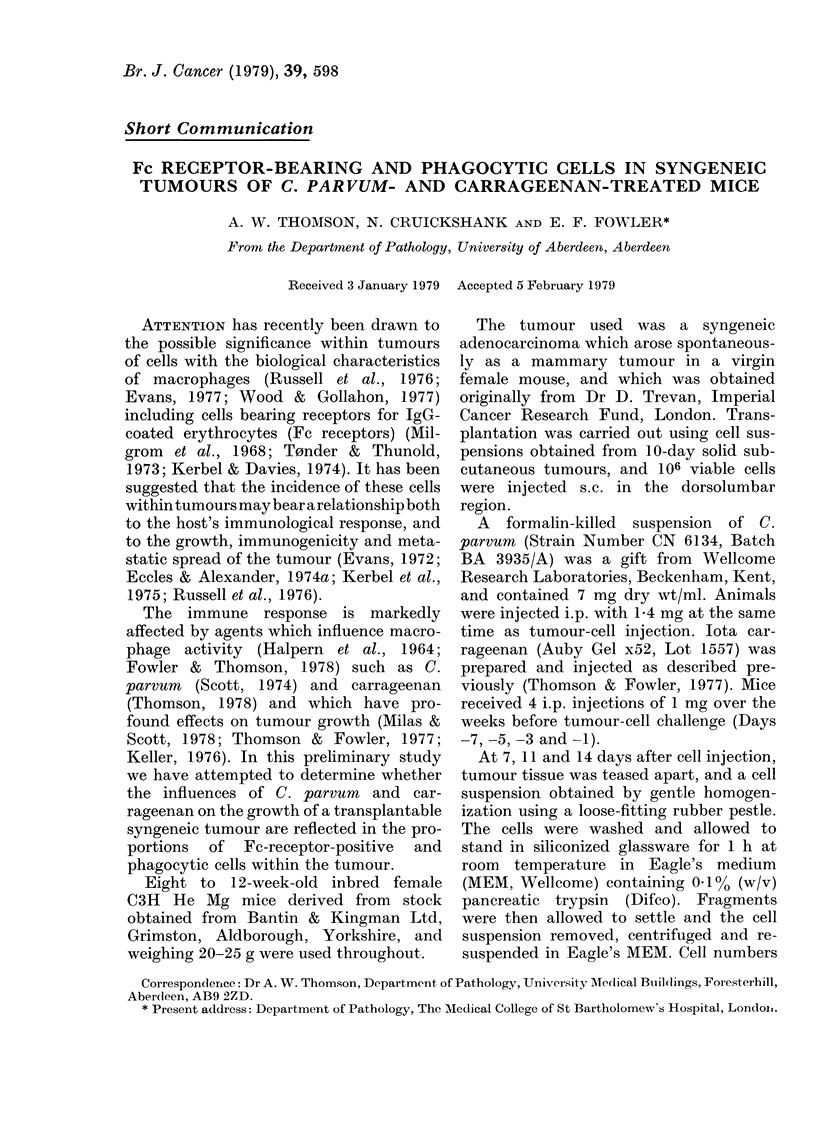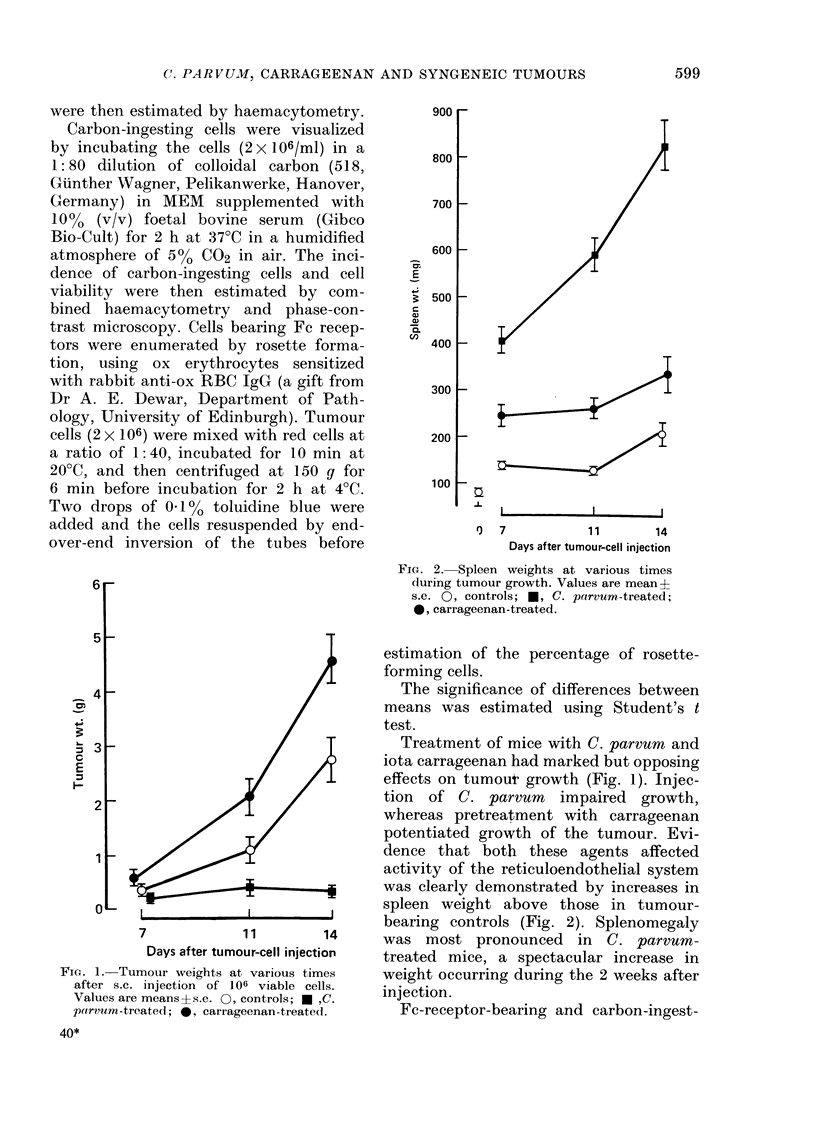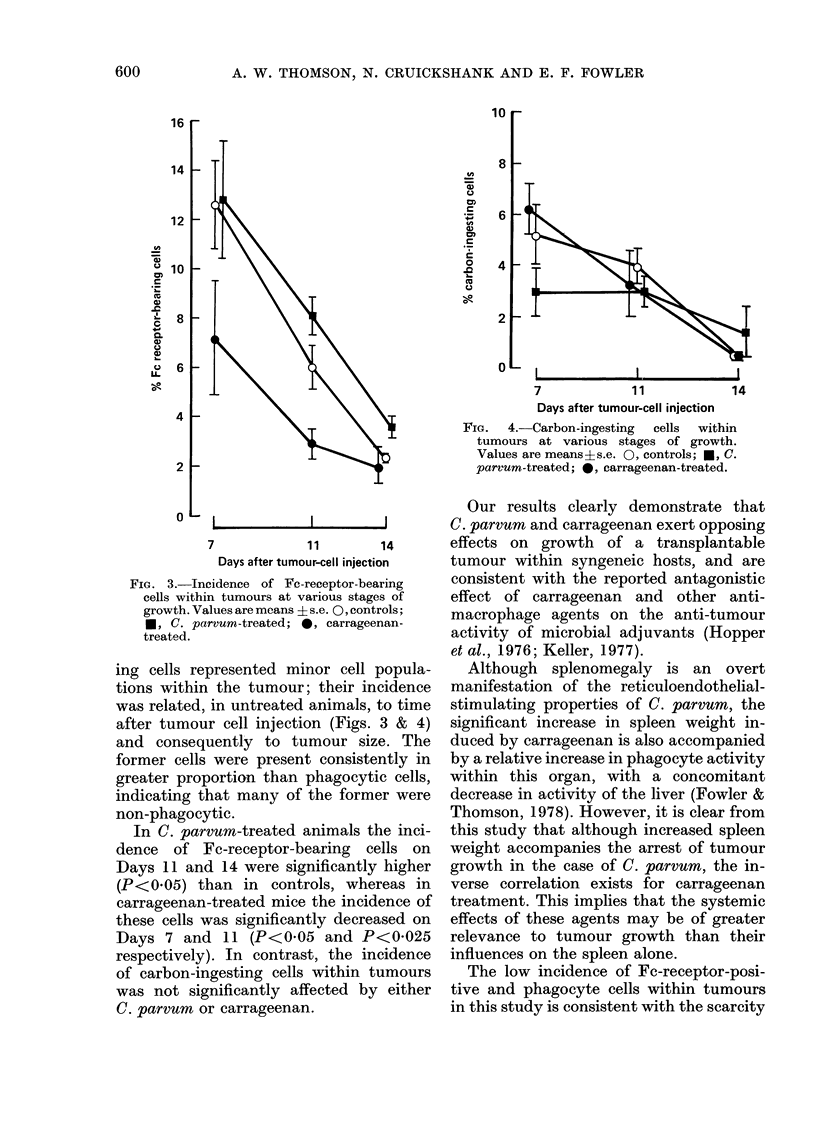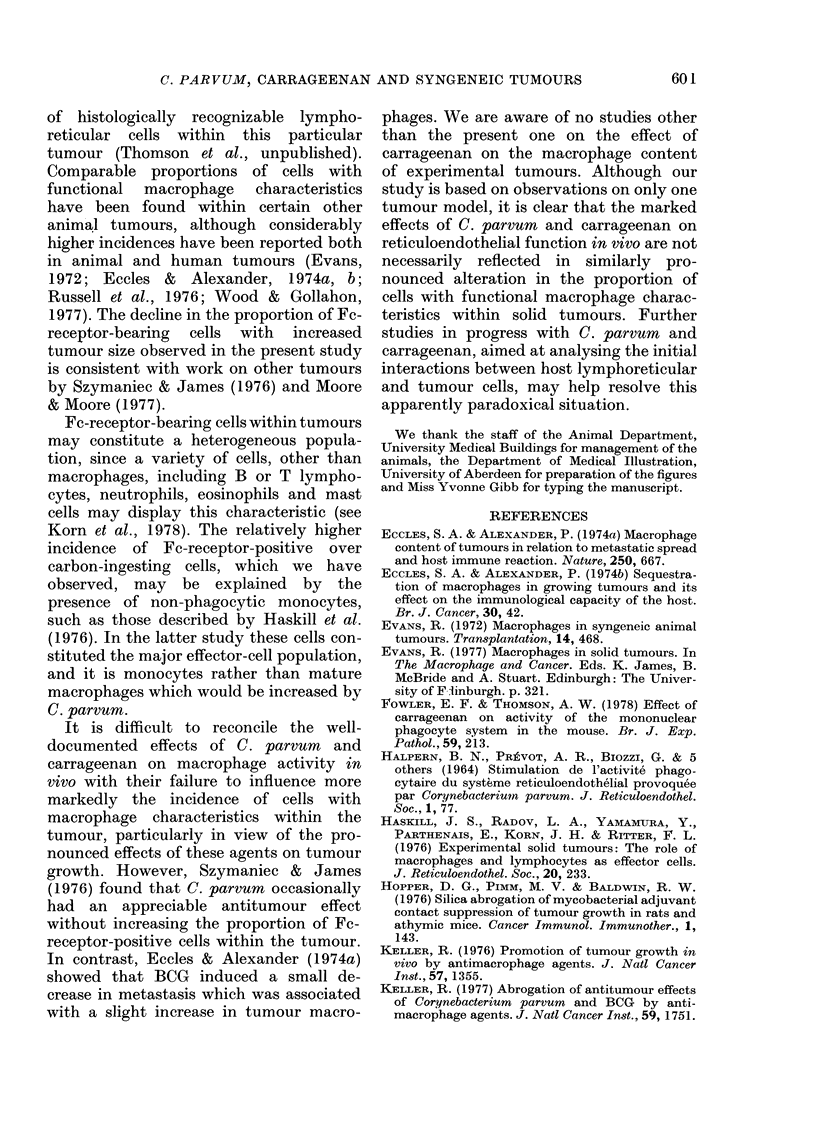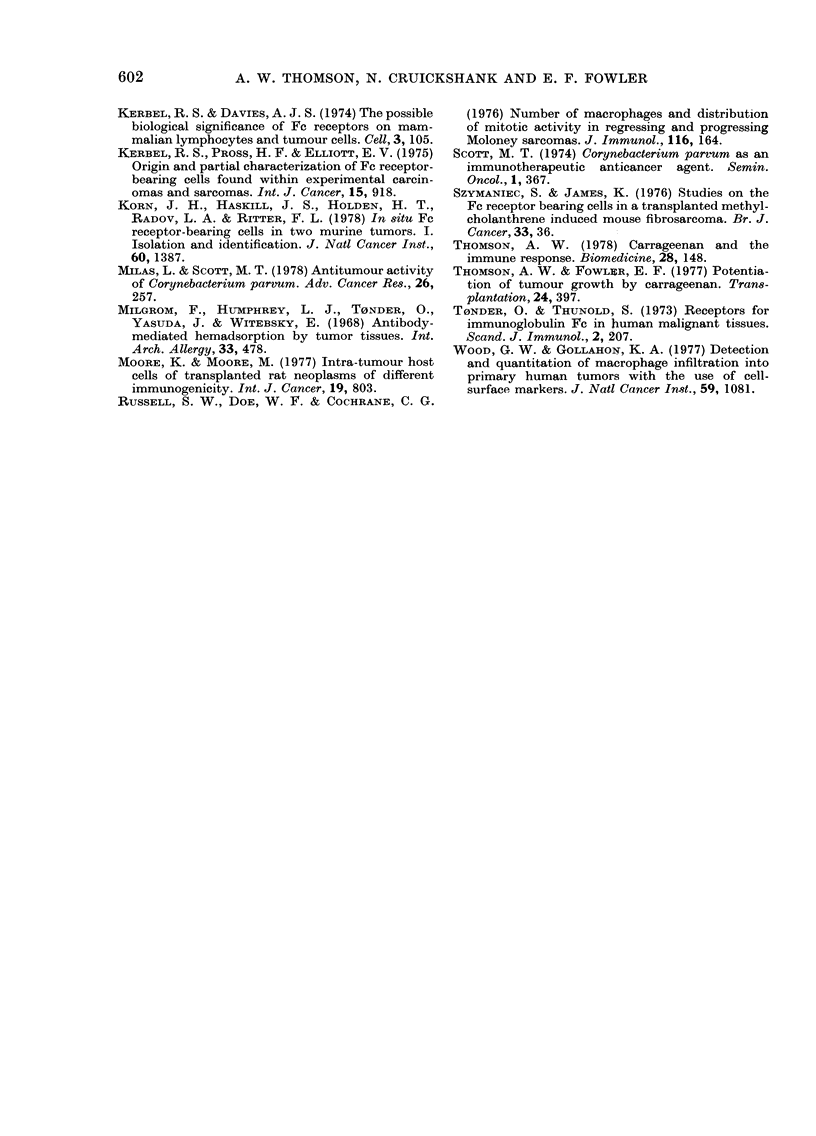# Fc receptor-bearing and phagocytic cells in syngeneic tumours of C. parvum- and carrageenan-treated mice.

**DOI:** 10.1038/bjc.1979.109

**Published:** 1979-05

**Authors:** A. W. Thomson, N. Cruickshank, E. F. Fowler


*Br. J. Cancer* (1979), **39**, 598

***Short Communication***

### Fc RECEPTOR-BEARING AND PHAGOCYTIC CELLS IN SYNGENEIC TUMOURS OF *C. PARVUM*- AND CARRAGEENAN-TREATED MICE

A. W. THOMSON, N. CRUICKSHANK AND E. F. FOWLER*

*From the Department of Pathology, University of Aberdeen, Aberdeen*

Received 3 January 1979 Accepted 5 February 1979

ATTENTION has recently been drawn to the possible significance within tumours of cells with the biological characteristics of macrophages (Russell *et al.*, 1976; Evans, 1977; Wood & Gollahon, 1977) including cells bearing receptors for IgG-coated erythrocytes (Fc receptors) (Milgrom *et al.*, 1968; Tønder & Thunold, 1973; Kerbel & Davies, 1974). It has been suggested that the incidence of these cells within tumours may bear a relationship both to the host's immunological response, and to the growth, immunogenicity and metastatic spread of the tumour (Evans, 1972; Eccles & Alexander, 1974*a*; Kerbel *et al.*, 1975; Russell *et al.*, 1976).

The immune response is markedly affected by agents which influence macrophage activity (Halpern *et al.*, 1964; Fowler & Thomson, 1978) such as *C. parvum* (Scott, 1974) and carrageenan (Thomson, 1978) and which have profound effects on tumour growth (Milas & Scott, 1978; Thomson & Fowler, 1977; Keller, 1976). In this preliminary study we have attempted to determine whether the influences of *C. parvum* and carrageenan on the growth of a transplantable syngeneic tumour are reflected in the proportions of Fc-receptor-positive and phagocytic cells within the tumour.

Eight to 12-week-old inbred female C3H He Mg mice derived from stock obtained from Bantin & Kingman Ltd, Grimston, Aldborough, Yorkshire, and weighing 20–25 g were used throughout.

The tumour used was a syngeneic adenocarcinoma which arose spontaneously as a mammary tumour in a virgin female mouse, and which was obtained originally from Dr D. Trevan, Imperial Cancer Research Fund, London. Transplantation was carried out using cell suspensions obtained from 10-day solid subcutaneous tumours, and $10^6$ viable cells were injected s.c. in the dorsolumbar region.

A formalin-killed suspension of *C. parvum* (Strain Number CN 6134, Batch BA 3935/A) was a gift from Wellcome Research Laboratories, Beckenham, Kent, and contained 7 mg dry wt/ml. Animals were injected i.p. with 1·4 mg at the same time as tumour-cell injection. Iota carrageenan (Auby Gel x52, Lot 1557) was prepared and injected as described previously (Thomson & Fowler, 1977). Mice received 4 i.p. injections of 1 mg over the weeks before tumour-cell challenge (Days −7, −5, −3 and −1).

At 7, 11 and 14 days after cell injection, tumour tissue was teased apart, and a cell suspension obtained by gentle homogenization using a loose-fitting rubber pestle. The cells were washed and allowed to stand in siliconized glassware for 1 h at room temperature in Eagle's medium (MEM, Wellcome) containing 0·1% (w/v) pancreatic trypsin (Difco). Fragments were then allowed to settle and the cell suspension removed, centrifuged and resuspended in Eagle's MEM. Cell numbers

Correspondence: Dr A. W. Thomson, Department of Pathology, University Medical Buildings, Foresterhill, Aberdeen, AB9 2ZD.

* Present address: Department of Pathology, The Medical College of St Bartholomew's Hospital, London.

were then estimated by haemacytometry.

Carbon-ingesting cells were visualized by incubating the cells ($2 \times 10^6$/ml) in a 1:80 dilution of colloidal carbon (518, Günther Wagner, Pelikanwerke, Hanover, Germany) in MEM supplemented with 10% (v/v) foetal bovine serum (Gibco Bio-Cult) for 2 h at 37°C in a humidified atmosphere of 5% $CO_2$ in air. The incidence of carbon-ingesting cells and cell viability were then estimated by combined haemacytometry and phase-contrast microscopy. Cells bearing Fc receptors were enumerated by rosette formation, using ox erythrocytes sensitized with rabbit anti-ox RBC IgG (a gift from Dr A. E. Dewar, Department of Pathology, University of Edinburgh). Tumour cells ($2 \times 10^6$) were mixed with red cells at a ratio of 1:40, incubated for 10 min at 20°C, and then centrifuged at 150 *g* for 6 min before incubation for 2 h at 4°C. Two drops of 0·1% toluidine blue were added and the cells resuspended by end-over-end inversion of the tubes before

FIG. 1.—Tumour weights at various times after s.c. injection of $10^6$ viable cells. Values are means ± s.e. ○, controls; ■ ,*C. parvum*-treated; ●, carrageenan-treated.

FIG. 2.—Spleen weights at various times during tumour growth. Values are mean ± s.e. ○, controls; ■, *C. parvum*-treated; ●, carrageenan-treated.

estimation of the percentage of rosette-forming cells.

The significance of differences between means was estimated using Student's *t* test.

Treatment of mice with *C. parvum* and iota carrageenan had marked but opposing effects on tumour growth (Fig. 1). Injection of *C. parvum* impaired growth, whereas pretreatment with carrageenan potentiated growth of the tumour. Evidence that both these agents affected activity of the reticuloendothelial system was clearly demonstrated by increases in spleen weight above those in tumour-bearing controls (Fig. 2). Splenomegaly was most pronounced in *C. parvum*-treated mice, a spectacular increase in weight occurring during the 2 weeks after injection.

Fc-receptor-bearing and carbon-ingest-

FIG. 3.—Incidence of Fc-receptor-bearing cells within tumours at various stages of growth. Values are means ±s.e. ○, controls; ■, *C. parvum*-treated; ●, carrageenan-treated.

FIG. 4.—Carbon-ingesting cells within tumours at various stages of growth. Values are means±s.e. ○, controls; ■, *C. parvum*-treated; ●, carrageenan-treated.

ing cells represented minor cell populations within the tumour; their incidence was related, in untreated animals, to time after tumour cell injection (Figs. 3 & 4) and consequently to tumour size. The former cells were present consistently in greater proportion than phagocytic cells, indicating that many of the former were non-phagocytic.

In *C. parvum*-treated animals the incidence of Fc-receptor-bearing cells on Days 11 and 14 were significantly higher ($P<0{\cdot}05$) than in controls, whereas in carrageenan-treated mice the incidence of these cells was significantly decreased on Days 7 and 11 ($P<0{\cdot}05$ and $P<0{\cdot}025$ respectively). In contrast, the incidence of carbon-ingesting cells within tumours was not significantly affected by either *C. parvum* or carrageenan.

Our results clearly demonstrate that *C. parvum* and carrageenan exert opposing effects on growth of a transplantable tumour within syngeneic hosts, and are consistent with the reported antagonistic effect of carrageenan and other anti-macrophage agents on the anti-tumour activity of microbial adjuvants (Hopper *et al.*, 1976; Keller, 1977).

Although splenomegaly is an overt manifestation of the reticuloendothelial-stimulating properties of *C. parvum*, the significant increase in spleen weight induced by carrageenan is also accompanied by a relative increase in phagocyte activity within this organ, with a concomitant decrease in activity of the liver (Fowler & Thomson, 1978). However, it is clear from this study that although increased spleen weight accompanies the arrest of tumour growth in the case of *C. parvum*, the inverse correlation exists for carrageenan treatment. This implies that the systemic effects of these agents may be of greater relevance to tumour growth than their influences on the spleen alone.

The low incidence of Fc-receptor-positive and phagocyte cells within tumours in this study is consistent with the scarcity of histologically recognizable lymphoreticular cells within this particular tumour (Thomson *et al.*, unpublished). Comparable proportions of cells with functional macrophage characteristics have been found within certain other animal tumours, although considerably higher incidences have been reported both in animal and human tumours (Evans, 1972; Eccles & Alexander, 1974*a*, *b*; Russell *et al.*, 1976; Wood & Gollahon, 1977). The decline in the proportion of Fc-receptor-bearing cells with increased tumour size observed in the present study is consistent with work on other tumours by Szymaniec & James (1976) and Moore & Moore (1977).

Fc-receptor-bearing cells within tumours may constitute a heterogeneous population, since a variety of cells, other than macrophages, including B or T lymphocytes, neutrophils, eosinophils and mast cells may display this characteristic (see Korn *et al.*, 1978). The relatively higher incidence of Fc-receptor-positive over carbon-ingesting cells, which we have observed, may be explained by the presence of non-phagocytic monocytes, such as those described by Haskill *et al.* (1976). In the latter study these cells constituted the major effector-cell population, and it is monocytes rather than mature macrophages which would be increased by *C. parvum*.

It is difficult to reconcile the well-documented effects of *C. parvum* and carrageenan on macrophage activity *in vivo* with their failure to influence more markedly the incidence of cells with macrophage characteristics within the tumour, particularly in view of the pronounced effects of these agents on tumour growth. However, Szymaniec & James (1976) found that *C. parvum* occasionally had an appreciable antitumour effect without increasing the proportion of Fc-receptor-positive cells within the tumour. In contrast, Eccles & Alexander (1974*a*) showed that BCG induced a small decrease in metastasis which was associated with a slight increase in tumour macrophages. We are aware of no studies other than the present one on the effect of carrageenan on the macrophage content of experimental tumours. Although our study is based on observations on only one tumour model, it is clear that the marked effects of *C. parvum* and carrageenan on reticuloendothelial function *in vivo* are not necessarily reflected in similarly pronounced alteration in the proportion of cells with functional macrophage characteristics within solid tumours. Further studies in progress with *C. parvum* and carrageenan, aimed at analysing the initial interactions between host lymphoreticular and tumour cells, may help resolve this apparently paradoxical situation.

We thank the staff of the Animal Department, University Medical Buildings for management of the animals, the Department of Medical Illustration, University of Aberdeen for preparation of the figures and Miss Yvonne Gibb for typing the manuscript.

### REFERENCES

ECCLES, S. A. & ALEXANDER, P. (1974*a*) Macrophage content of tumours in relation to metastatic spread and host immune reaction. *Nature*, **250**, 667.

ECCLES, S. A. & ALEXANDER, P. (1974*b*) Sequestration of macrophages in growing tumours and its effect on the immunological capacity of the host. *Br. J. Cancer*, **30**, 42.

EVANS, R. (1972) Macrophages in syngeneic animal tumours. *Transplantation*, **14**, 468.

EVANS, R. (1977) Macrophages in solid tumours. In *The Macrophage and Cancer*. Eds. K. James, B. McBride and A. Stuart. Edinburgh: The University of Edinburgh. p. 321.

FOWLER, E. F. & THOMSON, A. W. (1978) Effect of carrageenan on activity of the mononuclear phagocyte system in the mouse. *Br. J. Exp. Pathol.*, **59**, 213.

HALPERN, B. N., PRÉVOT, A. R., BIOZZI, G. & 5 others (1964) Stimulation de l'activité phagocytaire du système reticuloendothélial provoquée par *Corynebacterium parvum*. *J. Reticuloendothel. Soc.*, **1**, 77.

HASKILL, J. S., RADOV, L. A., YAMAMURA, Y., PARTHENAIS, E., KORN, J. H. & RITTER, F. L. (1976) Experimental solid tumours: The role of macrophages and lymphocytes as effector cells. *J. Reticuloendothel. Soc.*, **20**, 233.

HOPPER, D. G., PIMM, M. V. & BALDWIN, R. W. (1976) Silica abrogation of mycobacterial adjuvant contact suppression of tumour growth in rats and athymic mice. *Cancer Immunol. Immunother.*, **1**, 143.

KELLER, R. (1976) Promotion of tumour growth *in vivo* by antimacrophage agents. *J. Natl Cancer Inst.*, **57**, 1355.

KELLER, R. (1977) Abrogation of antitumour effects of *Corynebacterium parvum* and BCG by antimacrophage agents. *J. Natl Cancer Inst.*, **59**, 1751.

KERBEL, R. S. & DAVIES, A. J. S. (1974) The possible biological significance of Fc receptors on mammalian lymphocytes and tumour cells. *Cell*, **3**, 105.

KERBEL, R. S., PROSS, H. F. & ELLIOTT, E. V. (1975) Origin and partial characterization of Fc receptor-bearing cells found within experimental carcinomas and sarcomas. *Int. J. Cancer*, **15**, 918.

KORN, J. H., HASKILL, J. S., HOLDEN, H. T., RADOV, L. A. & RITTER, F. L. (1978) *In situ* Fc receptor-bearing cells in two murine tumors. I. Isolation and identification. *J. Natl Cancer Inst.*, **60**, 1387.

MILAS, L. & SCOTT, M. T. (1978) Antitumour activity of *Corynebacterium parvum*. *Adv. Cancer Res.*, **26**, 257.

MILGROM, F., HUMPHREY, L. J., TØNDER, O., YASUDA, J. & WITEBSKY, E. (1968) Antibody-mediated hemadsorption by tumor tissues. *Int. Arch. Allergy*, **33**, 478.

MOORE, K. & MOORE, M. (1977) Intra-tumour host cells of transplanted rat neoplasms of different immunogenicity. *Int. J. Cancer*, **19**, 803.

RUSSELL, S. W., DOE, W. F. & COCHRANE, C. G. (1976) Number of macrophages and distribution of mitotic activity in regressing and progressing Moloney sarcomas. *J. Immunol.*, **116**, 164.

SCOTT, M. T. (1974) *Corynebacterium parvum* as an immunotherapeutic anticancer agent. *Semin. Oncol.*, **1**, 367.

SZYMANIEC, S. & JAMES, K. (1976) Studies on the Fc receptor bearing cells in a transplanted methylcholanthrene induced mouse fibrosarcoma. *Br. J. Cancer*, **33**, 36.

THOMSON, A. W. (1978) Carrageenan and the immune response. *Biomedicine*, **28**, 148.

THOMSON, A. W. & FOWLER, E. F. (1977) Potentiation of tumour growth by carrageenan. *Transplantation*, **24**, 397.

TØNDER, O. & THUNOLD, S. (1973) Receptors for immunoglobulin Fc in human malignant tissues. *Scand. J. Immunol.*, **2**, 207.

WOOD, G. W. & GOLLAHON, K. A. (1977) Detection and quantitation of macrophage infiltration into primary human tumors with the use of cell-surface markers. *J. Natl Cancer Inst.*, **59**, 1081.